# Forensic Analysis of Umbilical and Newborn Blood Gas Values for Infants at Risk of Cerebral Palsy

**DOI:** 10.3390/jcm10081676

**Published:** 2021-04-14

**Authors:** Michael G. Ross

**Affiliations:** 1 Department of Obstetrics and Gynecology, Geffen School of Medicine at UCLA, Torrance, CA 90509, USA; mikeross@ucla.edu; 2 Department of Community Health Sciences, Fielding School of Public Health at UCLA, Torrance, CA 90509, USA; 3 Institute for Women’s and Children’s Health, The Lundquist Institute at Harbor-UCLA Medical Center, Torrance, CA 90509, USA

**Keywords:** acidosis, hypoxic-ischemic encephalopathy, cerebral palsy, base deficit

## Abstract

Cerebral palsy litigation cases account for the highest claims involving obstetricians/gynecologists, a specialty that ranks among the highest liability medical professions. Although epidemiologic studies indicate that only a small proportion of cerebral palsy (10–20%) is due to birth asphyxia, negligent obstetrical care is often alleged to be the etiologic factor, resulting in contentious medical-legal conflicts. Defense and plaintiff expert opinions regarding the etiology and timing of injury are often polarized, as there is a lack of established methodology for analysis. The objective results provided by umbilical cord and newborn acid/base and blood gas values and the established association with the incidence of cerebral palsy provide a basis for the forensic assessment of both the mechanism and timing of fetal neurologic injury. Using established physiologic and biochemical principles, a series of case examples demonstrates how an unbiased expert assessment can aid in both conflict resolution and opportunities for clinical education.

## 1. Introduction

Whether the result of an occurrence during prenatal development or consequence of labor or the postnatal period, cerebral palsy is a devastating outcome. Despite the clinical incorporation of electronic fetal monitoring, the rates of cerebral palsy have not changed significantly since the early 1970s, averaging 2 per 1000 live births [1]. Among those born with mild impairment, survival duration does not differ significantly from that of the population; however, mortality increases, as expected, with increasing severity of impairment.

In recent decades, medical progress has shifted the mortality for those with severe cerebral palsy from childhood to early adulthood [2]. As greater numbers of adults survive with cerebral palsy, the prevalence of high-burden medical conditions and healthcare resource utilization has markedly increased [3,4]. Among surviving children, the annual Medicaid costs are greater than 15 times that of unaffected children [5], with increasing costs among those with co-occurring intellectual disability [6]. The projected increase in survival and the cost of care contribute importantly to settlements and judgments in medical/legal cases.

Although epidemiologic studies indicate that only a small proportion of cerebral palsy (10–20%) is due to birth asphyxia [7], negligent obstetrical care is often alleged to be the etiologic factor. Electronic fetal monitoring has spawned a birth injury litigation crisis, resulting in “lottery-like judgments against physicians” [8] in the United States with verdicts of up to $100 million.

The assessment of the timing and mechanism of injury is of increasing importance since it relates to both litigation claims and clinical education regarding opportunities for improvement. Forensic analysis of clinical factors may discriminate prenatal, intrapartum and postnatal etiologies while assessment of the timing of asphyxial injury may determine whether intrapartum events were “preventable”. Commonly, in cerebral palsy litigation cases, fetal monitor tracings are utilized by both plaintiff and defense experts as the basis for opinions. However, the sensitivity, specificity and reproducibility of opinions of electronic fetal monitoring are limited [8,9,10].

The American College of Obstetricians and Gynecologists has codified classification of fetal heart tracings with a three-tier assessment in which category 1 is normal and category 3 is abnormal. However, the broad criteria of a category 2 tracing (neither normal nor definitively abnormal) and its management are controversial [11,12].

In contrast to semi-objective assessments of fetal monitor tracings, umbilical cord and newborn blood analyses provide an objective assessment of the metabolic state of the infant. The American College of Obstetricians and Gynecologists states that a threshold level of metabolic acidosis (base deficit (BD) greater than or equal to 12 mmol/L) or a mixed respiratory and metabolic acidosis (pH less than 7.0) must be met for cerebral palsy to be consistent with an acute peripartum or intrapartum asphyxia event. Recent studies have emphasized that the incidence of cerebral palsy further increases with a BD greater than or equal to 20 mmol/L [13,14]. Among infants with a BD of 12–19.9 mmol/L, the rate of cerebral palsy ranges from 2.1 to 4.0%, though there is a marked increase to 33% at BD values of 20 mmol/L. Thus, the majority of asphyxiated infants do not develop cerebral palsy.

The objective nature of umbilical cord and newborn blood values and the established association with the incidence of cerebral palsy can provide a basis for the forensic assessment of both the mechanism and timing of the neurologic injury. For an infant who develops cerebral palsy as a result of acute intrapartum hypoxemia, an obstetric intervention must have occurred prior to the BD reaching 12 mmol/L to assuredly prevent the neurologic injury.

This paper will examine a series of obstetric cases to illustrate the forensic process by which cord blood gas analyses can contribute to the determination of the timing and causation of a fetal injury. It should be emphasized that in liability cases, analyses are performed in conjunction with all available clinical, laboratory and radiologic information. All case descriptions are based upon actual liability cases that were assessed for either defendants or plaintiffs, though demographic details and select values have been modified for confidentiality or illustrative purposes. Cases were settled prior to trial.

## 2. Methods

For the forensic analysis of umbilical and newborn blood gas values, there are a number of accepted principals.

1. **Base deficit changes in labor**: Previous reports have described the normal BD values prior to labor, changes with stages of labor and fetal heart rate decelerations, as well as normal blood gas values following a vaginal delivery [15,16]. Briefly, the normal fetal arterial BD averages 2 mmol/L prior to labor. The latent phase of labor has minimal change in fetal umbilical artery BD under normal conditions, though BD increases ~1 mmol/L every three-hours of the active phase and ~1 mmol/L per hour of active pushing in the second stage. Early decelerations do not increase fetal umbilical artery BD, though variable decelerations (dependent upon degree and duration) and late decelerations increase BD in a predictive manner [15,16,17,18,19,20,21] The duration of fetal bradycardia (50–70 bpm) corresponds to an increased BD by 1 mmol/L for every 2 minutes. The normal umbilical BD_blood_ values following vaginal delivery approximate 5–6 mmol/L for umbilical artery and 4–5 mmol/L for umbilical vein [22,23].
2. **Clearance of acid in utero**: Fetal acid clearance in utero is primarily across the placenta [24,25], as fetal renal blood flow is markedly reduced compared to postnatal values, and fetal renal acid clearance is immature [26]. As measured in animal models, placental clearance of metabolic acidosis occurs at a rate of ~0.1 mmol/L per minute during periods of normalized fetal blood flow and oxygenation [17,20,21]. Placental acid clearance may be limited under conditions of reduced placental function [19] or pathologies (i.e., placental abruption).
3. **BD changes during the neonatal period**: Under conditions of normal newborn transition or rapid resuscitation, newborns do not increase the level of acidosis from delivery values once spontaneous heart rate exceeds 100 bpm. If born with significant metabolic acidosis, newborns minimally clear acid during the first one to two hours of life due to the early life impairment of hepatic and renal metabolism and clearance mechanisms as well as inhibition by acidosis [27,28]. Ultimately, the ‘’clearance’’ of systemic lactic acidosis depends on the initial peak level and the return and maintenance of spontaneous circulation and appropriate oxygenation. As neonatal cardiac compression produces less than 50% of normal cardiac output [29], newborns experience increasing acidosis during cardiac resuscitation until spontaneous heart rate exceeds 100 bpm. Similarly, severe newborn anemia, hypotension or septic shock will increase neonatal BD.
4. **Use of BD extracellular fluid (ECF):** Blood gas analyzers measure pH, pCO_2_ and pO_2_, though bicarbonate values and BD are calculated. Most commercial blood gas analyzers report values as BD, though the calculation is performed for BD_blood._ However, BD_blood_ values are significantly increased by elevated levels of pCO_2_ that commonly occur in fetal umbilical artery samples and may be dramatically elevated in cases of end-labor bradycardia. Thus, whereas BD_blood_ values may be appropriate for most children and adults, recent reports have emphasized that BD_ECF_ [13,30] should be used rather than BD_blood_, as BD_ECF_ corrects for the altered pCO_2_ values. With the increasing recognition of BD_ECF,_ many of the commercial blood gas analyzers now include this measurement. Alternatively, formulas for the calculation of BD_ECF_ from measured pH and pCO_2_ are readily available. In this manuscript, the radiometer (Radiometer Medical, Brønshøj, Denmark) formula was utilized for all BD_ECF_ calculations [31,32].
5. **Comparison of umbilical artery and vein BD values:** Under normal conditions, the (commonly reported) umbilical arterial BD_blood_ is ~1 mmol/L greater than umbilical vein values [23,33], primarily due to the higher umbilical vein pCO_2_. BD_ECF_ values in the artery and vein are commonly similar as metabolic lactic acid is cleared slowly across the placenta. Under conditions of complete umbilical cord occlusion or placental separation from the uterine wall (complete placental abruption), there is no significant flow from the umbilical artery through the placenta to the umbilical vein. Consequently, umbilical venous values at birth represent fetal values at the time of the occurrence of complete cord occlusion or placental separation, while umbilical artery values represent the newborn at the time of birth [15], though the arterial level of acidosis may be impacted by prior occurrences of fetal ischemia or hypoxia. Fetal arterial BD increases by ~0.5 mmHg per minute of complete cord occlusion [20]. Thus, in conditions of complete cord occlusion umbilical venous blood can be completely normal, representing the state of the placenta prior to the sentinel event, though the fetus suffers from severe hypoxia-ischemia [34]. The difference between artery and vein values may be utilized to time the occurrence of cord occlusion or placental separation. Similarly, under conditions of complete umbilical cord occlusion, fetal umbilical artery pCO_2_ initially increases by ~7 mmHg per minute due to absent placental CO_2_ clearance [35].
6. **Umbilical artery and vein blood gas values:** On occasion, samples of umbilical artery and vein blood are obtained from the same vessel, or samples are mislabeled. Normal fetal umbilical arterial and venous O_2_ and CO_2_ values [34], in conjunction with pH and BD values, can be used to assess if the values are consistent with the identified vessel. Similarly, if blood samples are exposed to an air bubble, umbilical pO_2_ values will increase and pCO_2_ values decrease due to atmospheric concentrations.
7. **Additional criteria for timing hypoxic ischemic injury:** In addition to umbilical cord and newborn BD and blood gas values, Apgar scores, nucleated red blood cell count (nRBC), newborn platelet count and evidence of cerebral injury and edema may provide insight into timing. Briefly, as detailed in Neonatal Encephalopathy and Neurologic Outcome, Second Edition, a 5-min Apgar score of 7–10 is classified as reassuring, a score of 4–6 as moderately abnormal, and a score of 0–3 as low in the term infant and late-preterm infant [36]. Thus, 5-min Apgar scores of 7 or more are generally inconsistent with a sentinel hypoxic-ischemic event during labor. Whereas the normal nRBC count in a term infant is 0–4 nRBC/100 white blood cells [37], pre-existing hypoxic injury may produce nRBC counts of ≥26/100 white blood cells [38]. Acute hypoxic-ischemic injury typically results in an increase in nRBC counts, though to values below this level. Erythropoietin mediated stimulation requires ~ 24 h to increase newborn nRBC [39]. Thus, the relatively acute increase in nRBC following a sentinel obstetric event (e.g., uterine rupture) likely reflects release from fetal/newborn hematopoietic stores [40]. Nonspecific increases in nRBC counts may occur as a result of maternal smoking; anemia; hemolysis; and maternal diabetes [41]. Coagulopathy may be a consequence of asphyxia-induced hepatic dysfunction, as the international normalized ratio (INR) of asphyxiated infants may increase significantly on day 1 and 2 of life [42]. Newborn thrombocytopenia (<100,000) generally requires 24 h from a hypoxic injury to be manifest, while cerebral edema, sometimes evident by slit ventricles, generally does not present on an ultrasound or MRI (magnetic resonance imaging) until 18 to 24 h following an insult and generally abates by 5–6 days. Whereas deep gray matter (thalami, basal ganglia) injury is associated with an acute, profound hypoxic injury, cortical watershed regions have been associated with partial, prolonged hypoxic injury in term fetuses [43]. In addition to sites of injury, specific MRI sequences (T1, T2, diffusion weighted images, Flair and spectroscopy) [44] can differentiate myelination, ischemia, cytotoxic edema and other patterns of injury and can aid in timing. Early brain MRI showing ex vacuo brain changes suggests a more chronic timing in injury. Thus, an early brain MRI within the first one to two days of life may be valuable in the timing of an injury if it demonstrates a pattern of edema which would precede labor and delivery.
  Placental pathologic exams may also provide information regarding mechanism and timing of injury. Placental nRBC count reflects fetal blood nRBCs [45], and placental assessment of gross (e.g., abruptio) or microscopic [46] pathology may determine evidence of acute, subacute or chronic dysfunction.

## 3. Case Studies

**Case 1**: A 32-year-old, gravida 4, para 3 had prenatal care that was notable for intrauterine growth restriction, though with uterine artery Dopplers that fluctuated weekly from normal to high and back to normal resistance (systolic/diastolic) levels. The patient presented at 37 weeks in active labor and advanced cervical dilatation. The tracing on admission was an atypical pattern of oscillations with a baseline rate of 140 bpm. Within three-hours of admission, the patient progressed to complete dilation, and with the first maternal push, the fetal heart rate abruptly dropped and remained at a rate of 50–60 bpm. A vacuum delivery was performed, and the infant was delivered 20 min following the onset of bradycardia. A small for gestational age infant (2200 g) had Apgar scores of 0, 4 and 5 at 1, 5 and 10 min, respectively.

With resuscitation, a heart rate greater than 100 bpm was achieved at 4 min of age. The infant was noted to have a three-vessel cord, with a tight true knot in the cord. From the umbilical cord, only venous blood was obtained, demonstrating values of pH 7.10, pCO_2_ 49 mmHg, pO_2_ 35 mmHg and BD_blood_ 15 mmol/L. At one-hour of life, an arterial blood gas revealed a pH of 6.8, pCO_2_ 40 mmHg and pO_2_ 89 mmHg. Laboratory results within an hour of life revealed a nucleated red cell count of 75 per 100 white blood cells, and elevated PT and PTT. A cranial ultrasound performed at 8 h of life demonstrated evidence of cerebral edema. At one-month of age, the infant demonstrated an abnormal MRI with basal ganglia injury.

Analysis: The analysis of this case indicates an umbilical cord venous value that resulted in BD_ECF_ 12.9 mmol/L and a 1-hour arterial blood gas BD_ECF_ 25.2 mmol/L. The umbilical vein BD_ECF_ of 12.9 mmol/L (reflecting the fetal status prior to the bradycardia) suggests that the fetus already had significant acidosis prior to the onset of bradycardia. With 20 min of end-labor bradycardia, as a result of presumed complete cord occlusion due to the knot, the expected umbilical artery BD_ECF_ would be ~23 mmol/L. With a 4-minute resuscitation prior to the newborn heart rate of 100 bpm, the arterial BD_ECF_ would be 25 mmol/L. As the newborn does not significantly clear acid during the first hour of life, these values are consistent with the one-hour blood gas value BD_ECF_ 25.2 mmol/L.

The findings of an atypical fetal heart pattern on admission, markedly elevated newborn nRBC count, an early onset coagulopathy and ultrasound evidence of cerebral edema by 8 h are further evidence consistent with a hypoxic injury occurring prior to presentation at the hospital. Finding of a tight knot in the cord both explains the intrauterine growth restriction and the fluctuating umbilical artery Doppler values (with the cord knot intermittently tightening and loosening). Although the end-labor bradycardia contributed to the newborn acidosis, the patient likely presented to the hospital with a significant fetal acidosis due to a preexisting hypoxic-ischemic event associated with the true knot, after which the knot may have loosened, and ultimately tightened again as the fetus descended in the pelvis with maternal pushing and labor progression.

**Case 2:** A 38-year-old, gravida 2, para 1 presented at 41 weeks’ gestation in labor. An initial cervical exam was 4 cm dilatation and 90% effacement. Upon the initiation of fetal monitoring, the tracing demonstrated a rate of 140 to 150 bpm with moderate variability. At 6 cm dilatation, rupture of membranes revealed thin meconium, though thick meconium was observed at the time of vaginal delivery. Throughout the remaining labor, the fetus demonstrated intermittent variable decelerations, though continued periods of minimal and moderate variability. At delivery, an infant female weighing 3685 g had Apgar scores of 1, 5 and 7 at 1, 5 and 10 min, respectively. Chest compressions were initiated for 3 minutes until a spontaneous heart rate over 100 bpm was detected. There were no umbilical cord blood samples drawn. Due to neonatal respiratory compromise, an endotracheal tube placed at one-hour of life though pulse oximeter values were not recorded. At two-hours of life, a capillary blood gas revealed pH 6.75, pCO_2_ 129 mmHg, pO_2_ 41 mmHg and BD_blood_ 21.6 mmol/L, after which ventilator settings were modified. A chest X-ray revealed patchy infiltrates consistent with meconium aspiration syndrome. The infant subsequently developed encephalopathy and cerebral palsy.

Analysis: As occurs commonly, physicians may not always obtain umbilical cord blood samples from infants in the immediate newborn period. However, the lack of confirmed cord values may raise questions as to the effects of the intrapartum course. In such cases, newborn blood gas values can be of great value. In the present case, fetal monitoring through the time of delivery demonstrating continued periods of minimal and moderate heart rate variability (consistent with fetal quiet and active sleep cycles) suggests that this was not an intrapartum hypoxic ischemic injury. Alternating minimal and moderate heart rate variability is consistent with fetal quiet sleep/active sleep cycling. After birth, chest compressions were discontinued prior to the 5-minute Apgar score indicating a rapid resuscitation. Due to respiratory distress, the infant was intubated at one-hour of life, though the capillary blood gas revealed marked acidosis at two-hours of age. Notably, the BD_blood_ of 21.6 must be corrected for the markedly elevated capillary pCO_2_, resulting in a BD_ECF_ of 15.1. Together with BD values, the elevated newborn pCO_2_ and reduced pO_2_ values likely resulted from meconium aspiration causing inadequate oxygenation and ventilation and the severe acidosis. These findings are consistent with the occurrence of significant hypoxemia and acidosis post-delivery.

**Case 3:** A 22-year-old, gravida 2, para 0 presented at 41 weeks for a post-date induction. During the course of labor, she had periods of moderate variable decelerations and alternating mild and moderate variability. One hour into the second stage of labor, there was an acute loss of signal of the fetal heart rate. Attempts to find the fetal heart tones with the monitor doppler revealed only intermittent brief signals of 105 bpm. A decision was made for a cesarean and the infant delivered operatively 26 min later. An infant female with Apgar scores of 3, 3, 4 and 7 at 1, 5, 10 and 15 min, respectively, had a venous umbilical cord gas of pH 7.50, pCO_2_ 20 mmHg, pO_2_ 65 mmHg, BD_blood_ 7.3 mmol/L. The infant required cardiac compressions for 6 minutes, at which point the heart rate exceeded 100 bpm. An arterial blood gas was performed at 25 min of age, revealing pH 7.31, pCO_2_ 20 mmHg, pO_2_ mmHg, 139, BD_blood_ 14.7 mmol/L. The nRBC count was 10 per 100 white blood cells, and platelet count, PT and PTT were normal. The infant demonstrated hypoxic ischemic encephalopathy.

**Figure 1:**
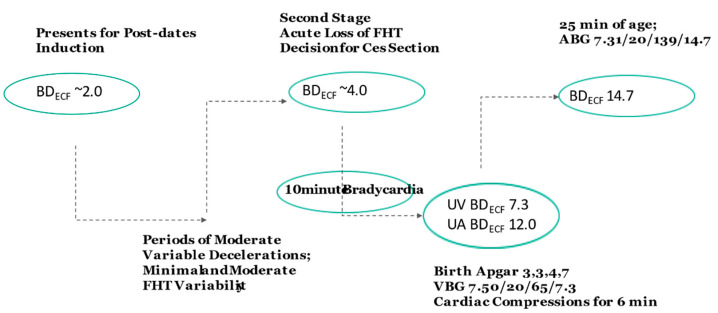
Flow Chart of Case 3. Values within ovals are determined by analysis (see text).

Analysis: The first step of analysis required inspection of the blood pH and gases. The venous gas at birth appeared to be contaminated or erroneous, as pH, pCO_2_ and pO_2_ values were not consistent with either physiologic umbilical artery or vein values. These values were consistent, however, with a venous sample exposed to an air bubble, either in the syringe or the blood gas analyzer. With air exposure, fetal pO_2_ values rose and pCO_2_ value dropped, and thus the pH rose. However, BD_ECF_ values did not change and the BD_ECF_ result could thus be utilized. With this understanding, the venous blood sample corrected to a BD_ECF_ 7.3 mmol/L (Figure 1), while the arterial blood gas at 25 min of age corrected to a BD_ECF_ 14.7 mmol/L. The venous blood sample confirmed that the fetus had relatively normal base deficit at the onset time of a bradycardia. Based on a 6-minute neonatal resuscitation, the newborn increased base deficit by 3 mmol/L during the first six minutes and then remained stable from an acid-base perspective until the arterial blood gas was performed. Thus, the predicted arterial base deficit at 6 minutes of life was 14.7 mmol/L, and the calculated umbilical artery BD_ECF_ at delivery was ~11.7 mmol/L.

If one “assumes” a continuous bradycardia for 26 min prior to delivery due to the loss of tracing, the umbilical artery to vein BD difference would be ~13 mmol/L. However, in the present case the difference of 4.4 mmol/L (11.7 minus 7.3 mmol/L) indicated that the fetus had a ~9-minute persistent bradycardia just prior to delivery. It is likely that intermittent variable decelerations beginning with the loss of signal increased fetal BD from a normal second stage BD value of ~4 mmol/L to 7.3 mmol/L, at which time the bradycardia occurred. The intermittent Doppler heart rate was consistent with maternal heart rate. These values demonstrate that the fetus had normal acid base status up until the loss of the fetal tracing and presumed bradycardia. The nRBC count of 10/100 white blood cells is consistent with an acute hypoxic ischemic event that occurred in the second stage of labor.

**Case 4:** A 27-year-old, gravida 4, para 3 presented at 42 weeks in spontaneous labor. With an initial cervical exam of 6 cm dilatation and 100% effacement, she progressed rapidly to complete dilatation. The fetal monitor Doppler demonstrated a rate of 110 to 120 bpm with moderate variability and intermittent variable decelerations. With the first three pushes, the tracing demonstrated severe variable decelerations (decrease to ≤70 bpm, lasting ≥60 s) with minimal variability. Following a change in maternal position to left lateral, administration of oxygen and an intravenous fluid bolus, there was a break in the tracing continuity, after which the monitor demonstrated a baseline of 120 bpm, moderate variability and a pattern of accelerations during each maternal push. As the second stage progressed, the baseline increased to 140 to 150 bpm with scattered breaks in the tracings, through continued accelerations with pushing. Following a 1 h 40 min second stage, an infant delivered spontaneously with meconium staining and Apgar scores of 1, 2 and 4 at 1, 5 and 10 min, respectively. Umbilical artery blood revealed pH 6.93, pCO_2_ 55 mmHg, pO_2_ 18 mmHg, BD_ECF_ 18.5 mmol/L and umbilical vein blood demonstrated pH 7.00, pCO_2_ 46 mmHg, pO_2_ 27 mmHg, BD_ECF_ 17.7 mmol/L. The infant suffered hypoxic ischemic encephalopathy and despite head cooling was ultimately diagnosed with cerebral palsy.

Analysis: This case represents a situation where the fetal heart monitor shifted from a fetal to maternal (uterine artery) tracing. The change from severe variable decelerations with minimal variability to moderate variability and accelerations coincident with maternal pushing is highly suggestive of maternal heart rate recording. The increase in baseline is consistent with second-stage maternal heart rates [47]. Notably, fetal heart rate accelerations are uncommon during maternal pushes.

The blood gases can provide insight into when the fetal BD exceeded a potential injury threshold of 12 mmol/L. The fetus normally enters the second stage with arterial BD 4 mmol/L. Severe variable decelerations increase BD by 0.5 mmol/L per minute [17,20], while placental BD clearance occurs at a rate of 0.1 mmol/L per minute. With four pushes every 10 min, repetitive severe variable decelerations would increase BD by 1.4 mmol/L every 10 min. The second stage of 100 min is thus consistent with the umbilical artery BD_ECF_ 18.5 mmol/L. The similarity of umbilical artery and vein BD_ECF_ values indicates there was not a predelivery persistent complete cord occlusion and bradycardia. Assuming the pattern of pushing (4 per 10 min) and severe variables continued throughout the second stage, the fetus likely exceeded BD 12 mmol/L at ~60 min into the second stage and thus would have had to be delivered prior to this time to assuredly prevent neurologic injury [17].

## 4. Discussion

In medical malpractice cases, both plaintiff and defense experts often opine on standard of care and liability. Although ideally based upon clinical standards and guidelines published by societies, institutions or academic centers [48], standards may be opined as based upon the expert’s “education and training” as well as his/her common practice. Unfortunately, the reliability of expert testimony may be arbitrary, with studies demonstrating limited or at best moderate agreement among experts within both academic and teaching hospital settings [49,50]. Furthermore, a knowledge of outcome may introduce bias as to judgement of clinical negligence [51]. An emphasis on evidence-based medicine has been proposed [52], but there are limited consensus guidelines or medical expert exams or certification procedures.

Even more challenging than liability questions, issues of causation and timing/preventability of injury typically require a knowledge base different from clinical practice standards. For fetal neurologic injury, a scientific knowledge of fetal, newborn and maternal physiology, acid-base dynamics, mechanisms of cellular injury, etc., is essential. Yet, studies of expert agreement on causality may be limited to yes/no choices [50], which are prone to assess association vs. causation [53]. Among the reports investigating the timing of cerebral palsy [54], select studies have proposed patterns of fetal monitor tracings which imply pre-labor hypoxic injuries [55,56]. Numerous reports have examined levels of lactate, pH and BD [14,57,58] in the prediction of intrapartum asphyxia and cerebral palsy. However, these reports have not provided an approach to specific timing of injury as described herein.

The four cases presented above provide examples of the forensic analysis of fetal injury. Case one is consistent with a pre-labor (i.e., preexisting) hypoxic injury, while case two illustrates the determination of a post-delivery (neonatal) hypoxic injury. Case three is an example as to how the umbilical artery and vein BD difference can predict the occurrence and duration of end-labor fetal bradycardia. Case four demonstrates how one can assess the timing of a hypoxic injury using the BD values at delivery, an assessment of the heart rate tracing and an understanding of the effects of stages of labor and heart rate decelerations on fetal BD.

Using the principles detailed in Methods, experts should attempt to remove potential bias, whether influenced by the knowledge of an adverse outcome or retention by plaintiff or defense attorneys. Some of the clinical criteria remain open to differences of opinion. For example, radiologic assessment of subtle brain edema or injury may result in subjective judgement, and fetal heart tracing interpretation has long been recognized as lacking consistency [59].

In contrast, umbilical cord and newborn blood gas values provide an objective data set by which to assess causation and timing. The expert, however, must properly correct BD_blood_ to BD_ECF_ values, recognize differences in arterial and venous values, and acknowledge potential alterations due to air exposure or liquid heparin coated syringes [60]. Knowledge of BD changes with stages of labor, heart rate decelerations and newborn transition may provide objective assessments that can aid in conflict resolution. Furthermore, levels of fetal acidosis may be estimated prospectively in labor, providing the clinician with an approximation of the BD and opportunities for modification of clinical practice. For example, second-stage labor with pushing-associated severe variable decelerations may be best managed by maternal pushing every other contraction, allowing adequate time for clearance of acidosis between each deceleration (i.e., ~0.1 mmol/L per min). Notably, therapeutic hypothermia has proven to be of value in the treatment of infants with severe intrapartum asphyxia [61]. These analyses should not alter the well-established criteria for hypothermic treatment.

The proposed approach of analysis has limitations in its reliance upon umbilical cord and/or newborn blood gases. Administration of bicarbonate to the newborn may chemically improve the BD, while significant maternal acidemia may increase fetal BD, unrelated to hypoxemia. This forensic analytic approach to timing of injury is intended to be used in conjunction with additional clinical variables, particularly the fetal monitor tracing. In addition, variables including Apgar scores, newborn clinical course, nRBC values and imaging studies are complementary and, as a general rule, the clinical factors should be consistent with the timing determined by BD analysis.

It is emphasized that these analyses address the timing and possible causation for the development of fetal/newborn hypoxemia and acidosis. For states or countries that have instituted programs of no-fault compensation for cerebral palsy [62], the proposed analysis of cases may provide opportunities for clinical practice modifications and clinical education. Medical-legal cases also hinge on very important aspects of standard of care, for which organizations (e.g., Society for Maternal Fetal Medicine, American Congress of Obstetricians and Gynecologists) may provide pertinent guidelines. When combined with established standard of care patterns, an objective assessment of causation and timing of injury can aid in the resolution of medical-legal cases.

## Data Availability

Not applicable.
